# ﻿Two new species of the millipede genus *Glyphiulus* Gervais, 1847 (Diplopoda, Spirostreptida, Cambalopsidae) from caves in northern Thailand

**DOI:** 10.3897/zookeys.1056.71395

**Published:** 2021-08-20

**Authors:** Natdanai Likhitrakarn, Sergei I. Golovatch, Sopark Jantarit

**Affiliations:** 1 Division of Plant Protection, Faculty of Agricultural Production, Maejo University, Chiang Mai, 50290, Thailand; 2 Biodiversity and Utilization Research Center of Maejo University, Maejo University, Chiang Mai, 50290, Thailand; 3 Institute for Problems of Ecology and Evolution, Russian Academy of Sciences, Leninsky pr. 33, Moscow 119071, Russia; 4 Excellence Center for Biodiversity of Peninsular Thailand, Faculty of Science, Prince of Songkla University, Hat Yai, Songkhla, 90110, Thailand

**Keywords:** Cave, diplopod fauna, *granulatus*-group, *javanicus*-group, key, map, subterranean habitat

## Abstract

Two new species of the genus *Glyphiulus* Gervais, 1847 are described and illustrated. The first species, *G.longus***sp. nov.**, is the second species of the *javanicus*-group to be found in Thailand. It resembles *G.guangnanensis* Jiang, Guo, Chen & Xie, 2018, from southern China, but is distinguished by a smaller size and the carinotaxic formula of the collum, combined with ♂ legs 1 bearing very strongly reduced telopodites, the anterior gonopods showing a pair of very long and slender apicomesal processes, and the denser plumose and stout flagella of the posterior gonopods. The second species, *G.promdami***sp. nov.**, the fifth member of the *granulatus*-group in Thailand, seems to be particularly similar to *G.subbedosae* Likhitrakarn, Golovatch & Panha, 2017, from Laos. However, it can be distinguished from the latter species mainly by showing a uniformly yellow collum and the posterior gonopod coxite bearing several strong setae in median and lateral views, coupled with the anterior gonopod coxosternum being microsetose in the anterior and medial parts in caudal view. An identification key to, and a distribution map of, all seven *Glyphiulus* species currently known to occur in Thailand are also provided.

## ﻿Introduction

The millipede family Cambalopsidae Cook, 1895 is very common, abundant and widespread in subterranean habitats of Thailand. Within this family, three genera are well-represented in Thai caves: *Glyphiulus* Gervais, 1847 (4 species), *Plusioglyphiulus* Silvestri, 1923 (14 species) and *Trachyjulus* Peters, 1864 (5 species) (Golovatch et al. 2009, 2011a, b, 2012b; Likhitrakarn et al. 2020). *Glyphiulus* is the most speciose and characteristic genus of the family Cambalopsidae (Golovatch et al. 2007a). Most *Glyphiulus* species are considered endemic to Southeast Asia and southern China, narrow endemism prevailing because, like most Diplopoda, their dispersal capacities are very limited (Golovatch et al. 2007b; Jiang et al. 2017, 2018; Likhitrakarn et al. 2017). Only two species, *G.granulatus* (Gervais, 1847), pantropical through numerous anthropochoric introductions (Golovatch et al. 2007a), and *G.javanicus* Carl, 1911, described from a sugar cane plantation in Java, Indonesia (Carl 1911), but from a still unclear area of origin lying somewhere in Indochina or China, are assumed to be truly widespread (Golovatch et al. 2007b, 2012a).

Two distinct species groups are currently recognized in *Glyphiulus*, based on the conformation of ♂ legs 1 (Golovatch et al. 2007a, b). The *granulatus*-group is distinguished by these legs usually being very strongly reduced to 1- or 2-segmented telopodite rudiments, coupled with two widely separated and curved prongs on the sternum. In the *javanicus*-group, ♂ legs 1 are usually with nearly normal 4- or 5-segmented telopodites, coupled with medially contiguous, but not entirely fused central coxal processes, and special carinotaxy patterns on the collum and following metaterga.

Five *Glyphiulus* species have hitherto been described from Thailand. The first species recorded was *G.siamensis* Mauriès, 1983, an epigean millipede from Doi Suthep, Chiang Mai (Mauriès 1983), which represents the *javanicus*-group (Golovatch et al., 2007a). All four following congeners have been reported from cave environments (Golovatch et al. 2011b) and they belong to the *granulatus*-group: *G.sattaa* Golovatch, Geoffroy, Mauriès & VandenSpiegel, 2011b from three caves (Tham Ku Kan (Ban Tham), Tham Nam Cham, and Tham Prah) in Mae Sai District, Chiang Rai Province; *G.duangdee* Golovatch, Geoffroy, Mauriès & VandenSpiegel, 2011b from Tham Chan, Khlong Trong National Park, Tong Saen Khan District, Uttaradit Province, *G.mongkon* Golovatch, Geoffroy, Mauriès & VandenSpiegel, 2011b from Tham Maho Lan, Ban Non Hin District, Loei Province, and *G.maidtreejit* Golovatch, Geoffroy, Mauriès & VandenSpiegel, 2011b from Tham Pha Hong, Lom Sak District, Phetchabun Province. All five species have narrow distributions and seem to be endemic to Thailand (Fig. 5 and Table 1).

In the present study, we describe two additional new species of *Glyphiulus* from northern Thailand. Furthermore, we provide a distributional map of, and a key to, all seven species of the genus currently known to occur in Thailand.

## ﻿Materials and methods

New material was collected in northern Thailand, in particular in limestone mountain and cave habitats. They were searched for and hand-collected using forceps. All populations were found to be sufficiently large and associated with bat guano in the twilight to deep and dark zones of the caves. The specimens taken were euthanized using a two-step method following AVMA Guidelines for the Euthanasia of Animals (AVMA 2013).

Specimens were then preserved in 95% ethanol for morphological and future molecular studies. All specimens were examined, measured, and photographed under a Nikon SMZ 745T trinocular stereo microscope, equipped with a Canon EOS 5DS R digital SLR camera. Digital images obtained were processed and edited with Adobe Photoshop CS6. Line drawings were based on photographs and examined under the stereo microscope equipped with a digital SLR camera.

Collecting sites were located by GPS using the WGS84 datum using a Garmin GPSMAP 60 CSx, and all coordinates and elevations were checked with Google Earth. The distribution maps of all *Glyphiulus* species recorded from Thailand were prepared using QGIS 3.18.0 (QGIS Development Team 2021). Google satellite maps were downloaded via the QuickMapServices plugin. The images were enhanced and arranged in plates with Adobe Photoshop CS6 software.

The terminology used and the carinotaxic formulae in the descriptions follow those in Golovatch et al. (2007a, b, 2009, 2011a, 2011b), while body segment counts are after Enghoff et al. (1993) and Golovatch et al. (2007a).

The holotypes, as well as most of the paratypes are housed in the Museum of Zoology, Chulalongkorn University (**CUMZ**), Bangkok, Thailand; some paratypes have been shared with the collections of the Zoological Museum, State University of Moscow, Russia (**ZMUM**) and the Princess Maha Chakri Sirindhorn Natural History Museum, Prince of Songkla University (**NHM-PSU**), Songkhla, Thailand, as indicated in the text.

## ﻿Taxonomy

**Family Cambalopsidae Cook, 1895**

### Genus *Glyphiulus* Gervais, 1847

#### ﻿ Glyphiulus longus sp. nov.

Taxon classificationAnimaliaDiplopodaCambalopsidae

C144C46A-E043-5268-BFD9-F9DB94F8D9DA

http://zoobank.org/B99F5675-C67F-4AA8-A777-6D987C2A975C

Figs 1 , 2

##### Type material.

***Holotype*** ♂: Thailand, Nan Province, Pon, Thung Chang District, Tham Nam Lod, 19°25'13"N, 101°04'15"E, ca 1420 m a.s.l., 30.05.2018, S. Jantarit leg.; CUMZ-CAM171. ***Paratypes***: 3 ♀: same locality as holotype; CUMZ-CAM171; 1 ♀: same locality as holotype; ZMUM; 1 ♀: same locality as holotype; NHM-PSU.

##### Name.

The species is so named to emphasize the long medial processes of the anterior and posterior gonopods; adjective.

##### Diagnosis.

**Figure 1.**
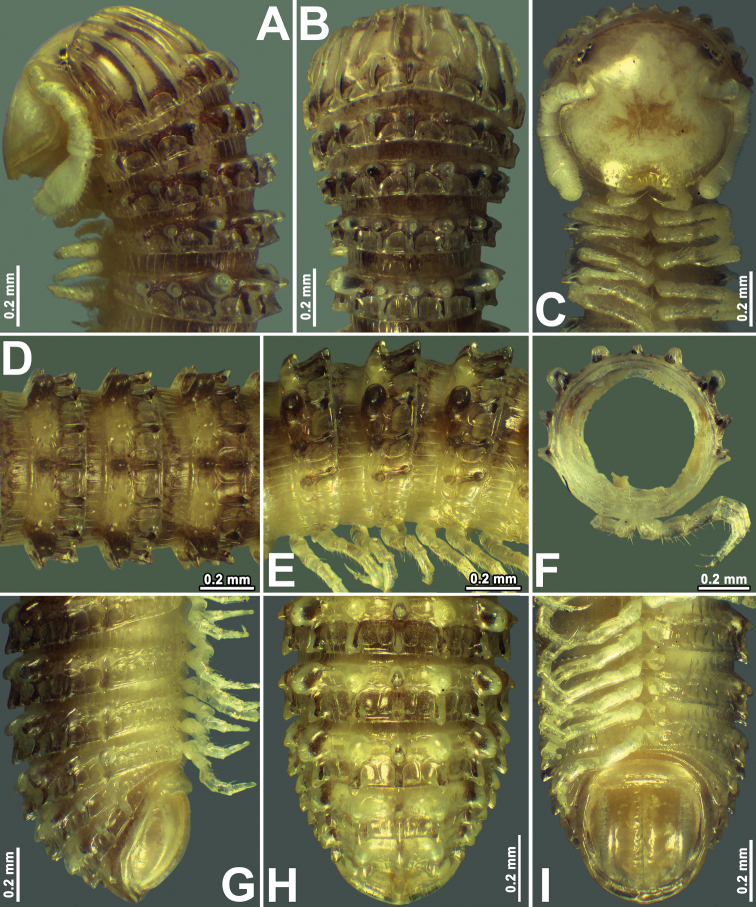
*Glyphiuluslongus* sp. nov., ♂ holotype **A–C** anterior part of body, lateral, dorsal, and ventral views, respectively **D, E** midbody rings, dorsal and lateral views, respectively **F** cross-section of a midbody ring **G–I** posterior part of body, lateral, dorsal, and ventral views, respectively.

**Figure 2.**
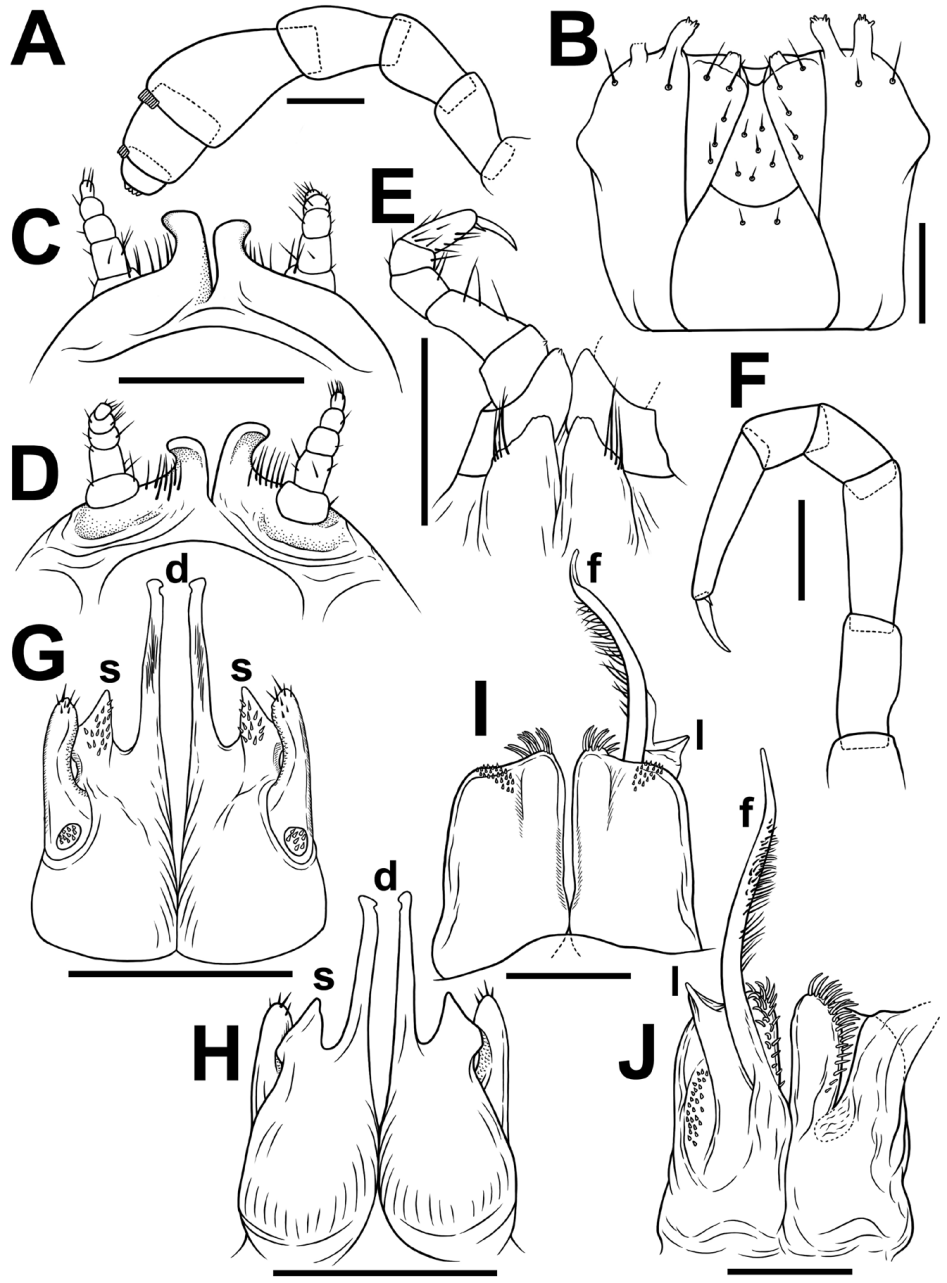
*Glyphiuluslongus* sp. nov., ♂ holotype **A** antenna, lateral view **B** gnathochilarium, ventral view **C, D** legs 1, oral and caudal views, respectively **E** legs 2, caudal view **F** midbody leg **G, H** anterior gonopods, caudal and oral views, respectively **I, J** posterior gonopods, oral and caudal views, respectively. Abbreviations: **d** apicomesal processes, **s** subsecuriform process, **f** flagellum process, **l** lamelliform lobe. Scale bar: 0.1 mm.

This new species seems to be particularly similar to *G.guangnanensis* Jiang, Guo, Chen & Xie, 2018, from Yunnan Province, China (Jiang et al. 2018), with which it shares the following diagnostic characters: very strong metatergal crests and unique carinotaxic formulae, coupled with certain anterior and posterior gonopod structural details. *Glyphiuluslongus* sp. nov. differs from *G.guangnanensis* by the smaller body size, 22–26 mm long (vs. larger, 38–54 mm long) and the carinotaxic formula of the collum: 1+2a+3c+4–5+6c+7a+pc+ma (vs. 1a+2c+3–4+5c+6a+pc+ma) (Fig. 1A, B), coupled with ♂ legs 1 showing very strongly reduced telopodites (Fig. 2C, D) (vs. nearly normal telopodites), the apicomesal processes (d) on the anterior gonopods being very long and slender (Fig. 2G, H) (vs. shorter and digitiform), and the presence of a very long, medially densely plumose and stout flagellum process (f) on the posterior gonopods (Fig. 2I, J) (vs. slim and smooth flagellum process).

##### Description.

Length of holotype ♂, 23.5 mm; that of paratypes, 22.8–26.5 mm (♀); midbody segments round in cross-section (Fig. 1F), their width (horizontal diameter) and height (vertical diameter) being similar; width of holotype ♂, 1.0 mm, of ♀ paratypes, 0.9–1.2 mm.

Coloration in alcohol (Fig. 1), after three years of preservation, uniformly red-brownish or dark castaneous brown to grey-brown, dorsal crests and porosteles usually dark brownish (Fig. 1A, B, D, E, H). Antennae and venter yellowish to pallid (Fig. 1A, C, E–G, I). Eyes blackish to brownish (Fig. 1A, C).

Body with 56p+2a+T rings/segments (♂ holotype); ♀ paratypes with 50–57p+2(1)a+T rings. Eye patches transversely ovoid, each composed of 2–5 rather flat ocelli in a single longitudinal row (Fig. 1A, C). Antennae short and clavate (Figs 1A, C, 2A), extending past ring 3 laterally, antennomeres 5 and 6 each with a small distoventral group or corolla of bacilliform sensilla (Fig. 2A). Gnathochilarium with a clearly separated promentum (Fig. 2B).

In width, head = ring 2 < collum = midbody rings (close to 13^th^ to 15^th^) < ring 4 = 5 < 7 = 3 < 8 = 10; body abruptly tapering towards telson on a few posteriormost rings (Fig. 1B). Postcollar constriction very evident (Fig. 1B).

Collum nearly smooth, carinotaxic formula 1+2a+3c+4–5+6c+7a+pc+ma (Fig. 1A–C), with 6+6 longitudinal crests starting from anterior edge, but both median crests interrupted in about caudal 2/3–3/4, being replaced there by similar 1+1+1 crests.

Following metaterga very strongly crested (Fig. 1A, B, D–H), especially from ring 5 on, whence porosteles commence (Fig. 1A, B), smaller tubercles in their stead on legless rings in front of telson due to loss of ozopores (Fig. 1G, H). Porosteles large, conical, round, directed caudolaterad, rather higher than wide (Fig. 1A, B, D–H). Midway metatergal crests on ring 5 distinctly divided into two at about 1/3 metatergal height, each half evident and well rounded, nearly undivided small tubercles in their stead in legless rings in front of telson (Fig. 1G, H). Carinotaxic formulae 2/2+I/i+3/3+I/i+2/2, all dorsal crests subdivided transversely and sharper, especially so lateral crests (Fig. 1B, D–H).

Tegument rather smooth, shining throughout (Fig. 1A, B, D–H). Fine longitudinal striations in front of stricture between pro- and metazonae, remaining surface of prozonae very delicately shagreened (Fig. 1D, E). Metatergal setae absent. Rings 2 and 3 each with long pleural flaps. Epiproct (Fig. 1G–I) simple, with a long dorsal crest in middle and small paramedian tubercles, regularly rounded caudally, faintly convex medially. Paraprocts regularly convex, each with premarginal sulci medially and a row of sparse setae at medial margin (Fig. 1I). Hypoproct transversely bean-shaped, slightly concave caudally, with 1+1 strongly separated setae near caudal margin (Fig. 1I).

Ventral flaps behind gonopod aperture on ♂ ring 7 barely distinguishable as low swellings forming no marked transverse ridge.

Legs short, on midbody rings about 2/3 length of body height (Figs 1A, C, E–G, 2F). Claw at base with a very small accessory spine about 1/6 the length of main claw (Fig. 2F).

♂ legs 1 highly characteristic (Fig. 2C, D) in being very strongly reduced, 5-segmented telopodites and a pair of large, subdigitiform, medially contiguous, but apically diverging coxal processes with groups of long and strong setae at base.

♂ legs 2 nearly normal (Fig. 2E), claw long and slender; only anteriorly, prefemur somewhat reduced, and femur abbreviated on oral face; penes broad, rounded, each with four or five strong setae distolaterally (Fig. 2E).

♂ legs 3 missing, lost.

Anterior gonopods (Fig. 2G, H) with a typical shield-like coxosternum, the latter modestly microsetose on caudal face (Fig. 2G), on each side with a very long, slender, chopstick-shaped, apicomesal process (d) and an evident subsecuriform process (s), tip narrowly rounded. Telopodite rather small, stout, movable, 1-segmented, lateral in position, with three or four strong apical setae and a field of microsetae at base (Fig. 2G, H), about as long as adjacent lateral corner of coxosternum, but lower than paramedian processes (d) of coxosternum.

Posterior gonopods (Fig. 2I, J) compact, broadly subquadrate, micropapillate medially on oral face; with a very long, medially densely plumose and stout flagellum process (f) (Fig. 2I, J); lamelliform lobe (l) higher than caudal piece of coxite, membranous, smooth, with an apical field of coniform microsetae laterally (Fig. 2J); each median and lateral part of coxite with dense, strong and curved setae (Fig. 2J).

##### Remarks.

This new species belongs to the *javanicus*-group which currently encompasses 32 described species (Golovatch et al. 2007b, 2011b, 2012a; Likhitrakarn et al. 2017; Jiang et al. 2017, 2018, 2020). The above new congener is the second one in this group to be reported from Thailand. Due to the absence of any potential morphological cave adaptations, i.e., of a longer body, of a lighter coloration, and of elongated femora and tarsi of walking legs (Liu et al. 2017), *G.longus* sp. nov. is only to be considered as troglophilic at most. The cave where the species was discovered is located at an elevation of ca 1420 m a.s.l. in Nan Province. The cave is rather narrow, hidden in a steep-sided doline in a riparian area. It consists of a streamway and a series of oxbow passages, with many links between these two parts to the cave. The areas where the cave was developed are surrounded by agricultural landscapes, i.e., cabbage and corn fields. The specimens were found on the cave floor with scattered bat guano and some debris brought in by the stream, ca 50 meters from the entrance. The temperature of the cave was 20.8–22.4 °C (mid-May), and the relative humidity was 85–90%. In the same cave and habitat, we also found Collembola (*Coecobrya* sp. and *Lepidocyrtus* sp.), Formicidae (*Ponera* sp.), Thelyphonida, mites and pseudoscorpions.

**Table 1.** Comparison of all seven *Glyphiulus* species known to occur in Thailand; n/a = no information.

Characters/Species	* G. duangdee *	*G.longus* sp. nov.	* G. maidtreejit *	* G. mongkon *	*G.promdami* sp. nov.	* G. sattaa *	* G. siamensis *
Group	* granulatus *	* javanicus *	* granulatus *	* granulatus *	* granulatus *	* granulatus *	* javanicus *
Length (mm)							
♂	14–22	23.5	22–24	12–20	14.5–18.4	23–40	23
♀	14–22	22.8–26.5	n/a	n/a	14.2–24.3	23–40	n/a
colour	uniformly brown to dark brown	uniformly red-brownish or dark castaneous brown to grey-brown, dorsal crests and porosteles usually dark brownish	dark grey-brown to dark brown, most of tergal crests/tubercles and ozopoiferous cones very dark brown	uniformly light yellow-brown	uniformly red-yellownish to grey-brown, dorsal crests and porosteles usually dark brownish	uniform light brown or brown, with tergal crests/tubercles and ozoporiferous cones dark brown	reddish brown
Eyes	8–13, blackish	2–5, blackish to brownish	14–16, blackish	6–8, light greyish	8–13, blackish to brownish	9–13, brown	n/a
Antennae	Long and moderately clavate; extending beyond segment 4 laterally	short and clavate, extending past ring 3 laterally	short and moderately clavate; extending beyond segment 3 laterally	short and moderately clavate; extending beyond segment 3 laterally	short and clavate, extending past ring 3 laterally	Long and moderately clavate; extending beyond segment 4 laterally	n/a
Body							
Holotype	52p+2a+T	56p+2a+T	52p+2a+T	31p+2a+T	58p+1a+T	58p+1a+T	53+2a+T
♂	40-56p+4-2a+T	n/a	46p+2a+T	31-43p+2a+T	35-58p+1–2a+T	44–72p+5-1a+T	n/a
♀	40-56p+4-2a+T	50–57p+2(1)a+T	n/a	31-43p+2a+T	39–51p+1-3a+T	44-72p+5-1a+T	n/a
Carinotaxy formula of collum	1a+2-5+pc+ma	1+2a+3c+4-5+6c+7a+pc+ma	1-6+7a+pc+ma	1-4+5a+pc+ma	1–6+7a+pc+ma	1-4+5a+6a+pc+ma	6/6+ma
Carinotaxy formula of midbody rings	3/3+I/i+4//3+I/i+3/3	2/2+I/i+3/3+I/i+2/2	(2)3/(2)3+I/i+4//3+I/i+3/3	3/3+I/i+4//3+I/i+3/	3/3+I/i+4/3+I/i+3/3	2/2+I/i+4/3(2)/3+I/i+2/2	3/3+I/i+3/3I/i+3/3
Ratio of leg length comparing to their ring height	0.7–0.8	0.67	0.9–1.0	1.1–1.2	0.5	1.0–1.1	n/a
Habitat	caves	caves	caves	caves	caves	caves	epigean
Locality	Tong Saen Khan District, Uttaradit	Thung Chang District, Nan	Lom Sak District, Phetchabun	Ban Non Hin District, Loei	Na Noi District and Na Muen District, Nan; Rong Kwang District, Phrae	Mae Sai District, Chiang Rai	Doi Suthep, Chiang Mai

#### ﻿ Glyphiulus promdami sp. nov.

Taxon classificationAnimaliaDiplopodaCambalopsidae

0A5069E6-6B5C-561B-BF28-2DD40A54F3E8

http://zoobank.org/31975FDC-B9BF-4130-9BE3-B06B9C0921EE

Figs 3 , 4

##### Type material.

***Holotype*** ♂: Thailand, Nan Province, Na Noi District, Tham Chetawan, 18°16'26"N, 100°34'43"E; 520 m a.s.l., 18.05.2018, S. Jantarit leg.; CUMZ-CAM169. ***Paratypes***: 5 ♂, 5 ♀: same locality as holotype; CUMZ-CAM169; 1 ♂, 1 ♀: same locality as holotype; ZMUM; 1 ♂, 1 ♀: same locality as holotype; NHM-PSU; 1 ♂, 3 juv.: same Province, Na Muen District, Tham La-ong, 18°14'18"N, 100°34'55"E, 648 m a.s.l., 18.05.2018, S. Jantarit leg.; CUMZ-CAM170; 3 ♂, 2 ♀: Phrae Province, Rong Kwang District, Tham Pha Phrai Wan, 18°25'18"N, 100°28'10"E, 419 m a.s.l., 17.05.2019, S. Jantarit leg.; CUMZ-CAM166.

##### Name.

The species is so named to honour Mr. Rueangrit Promdam, a carcinologist and researcher at the Princess Maha Chakri Sirindhorn Natural History Museum of Prince of Songkla University (NHM-PSU), who is interested in cave fauna in the country and who has collected many millipedes, including this new species, from various Thai caves.

##### Diagnosis.

**Figure 3.**
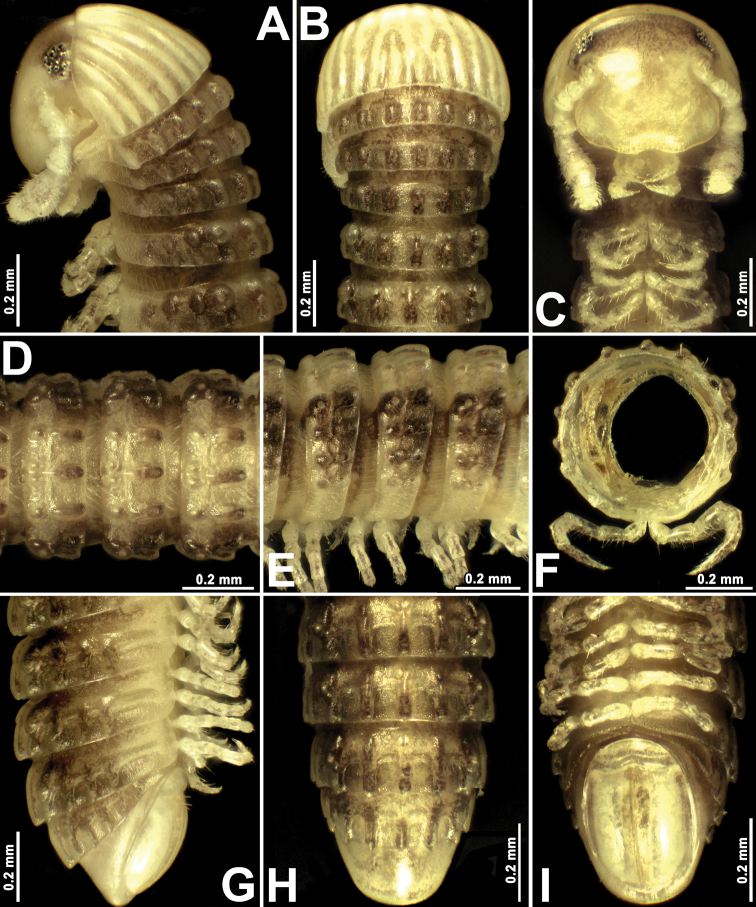
*Glyphiuluspromdami* sp. nov., ♂ paratype **A–C** anterior part of body, lateral, dorsal and ventral views, respectively **D, E** midbody rings, dorsal and lateral views, respectively **F** cross-section of a midbody ring **G–I** posterior part of body, lateral, dorsal and ventral views, respectively.

**Figure 4.**
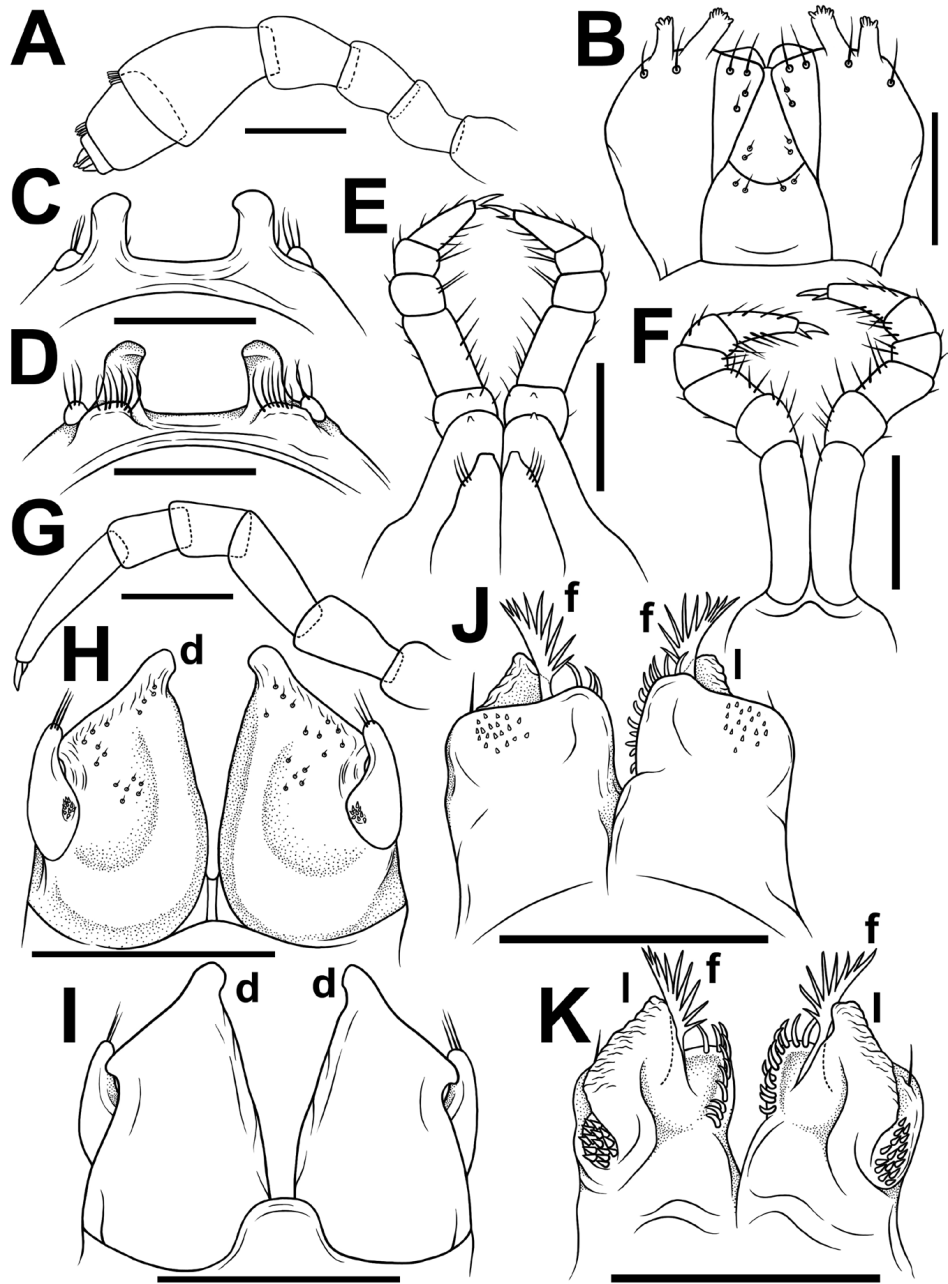
*Glyphiuluspromdami* sp. nov., ♂ holotype **A** antenna, lateral view **B** gnathochilarium, ventral view **C, D** legs 1, oral and caudal views, respectively **E** legs 2, caudal view **F** leg 3, caudal view **G** midbody leg **H, I** anterior gonopods, caudal and oral views, respectively **J, K** posterior gonopods, oral and caudal views, respectively. Abbreviations: **d** apicomesal processes, **f** flagellum process, **l** lamelliform lobe. Scale bar: 0.1 mm.

This new species seems to be particularly similar to *G.subbedosae* Likhitrakarn, Golovatch & Panha, 2017, from Laos (Likhitrakarn et al. 2017), with which it shares the following diagnostic characters: body size, colour pattern and unique carinotaxic formulae, coupled with certain anterior and posterior gonopod structural details. It differs from *G.subbedosae* primarily by a uniformly yellow collum (Fig. 3A–C) (vs. its anterior half darker) and the median and lateral parts of the posterior gonopod coxite with 10–12 strong setae (Fig. 4K) (vs. six strong setae), coupled with the anterior gonopod coxosternum being moderately microsetose in the anterior and medial parts on the caudal face (Fig. 4H) (vs. only in medial part).

##### Description.

**Figure 5.**
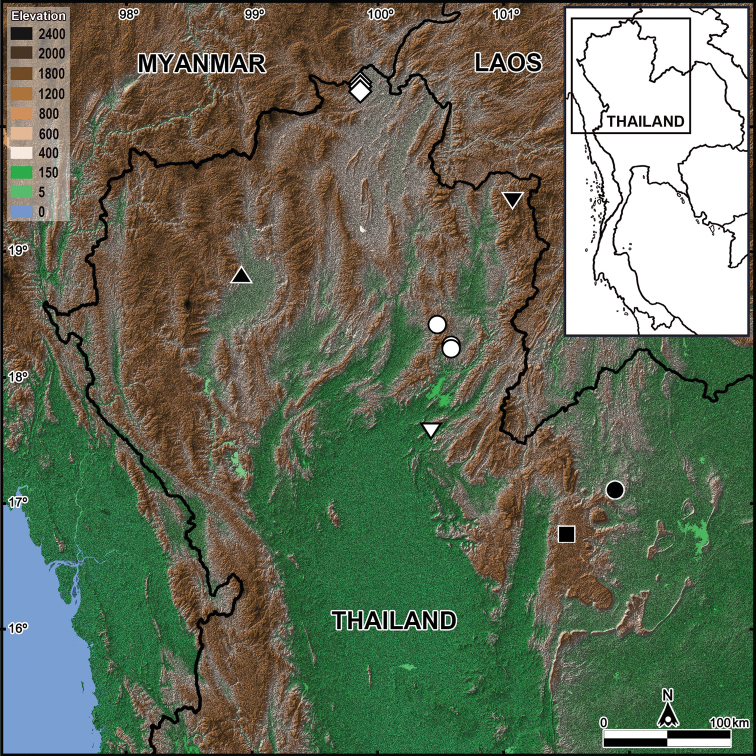
Distribution of *Glyphiulus* species in Thailand (seven species): Open diamond: *Glyphiulussattaa* Golovatch, Geoffroy, Mauriès & VandenSpiegel, 2011; Inverted filled triangle: *Glyphiuluslongus* sp. nov.; Filled triangle: *Glyphiulussiamensis* Mauriès, 1983; Open circle: *Glyphiuluspromdami* sp. nov.; Inverted open triangle: *Glyphiulusduangdee* Golovatch, Geoffroy, Mauriès & VandenSpiegel, 2011; Filled circle: *Glyphiulusmongkon* Golovatch, Geoffroy, Mauriès & VandenSpiegel, 2011; Filled square: *Glyphiulusmaidtreejit* Golovatch, Geoffroy, Mauriès & VandenSpiegel, 2011.

Length of holotype, 21.8 mm; adult paratypes 14.5–18.4 (♂) or 14.2–24.3 mm long (♀), juveniles 6.5–12.3 mm long; midbody rings round in cross-section (Fig. 5L), their width (horizontal diameter) and height (vertical diameter) being similar; width in holotype, 0.9 mm; in paratypes, 0.7–1.0 (♂), 0.8–1.1 (♀) or 0.5–0.7 mm (juveniles).

Coloration in alcohol (Fig. 3), after three years of preservation, uniformly red-yellownish to grey-brown, dorsal crests and porosteles usually dark brownish (Fig. 3A, B, D, E, H). Head, collum, antennae and venter yellowish to pallid (Fig. 3A, C, E–G, I). Eyes blackish to brownish (Fig. 3A, C).

Body with 58p+1a+T rings (♂ holotype); paratypes with 35–58p+1–2a+T (♂), 39–51p+1–3a+T (♀) or 25–35+2–5a+T (juveniles). Eye patches transversely ovoid, each composed of 8–13 blackish, rather flat ocelli in three or four irregular longitudinal rows (Fig. 3A). Antennae short and clavate (Figs 3A, C, 4A), extending past ring 3 laterally, antennomeres 5 and 6 each with a small distoventral group or corolla of bacilliform sensilla (Fig. 4A). Gnathochilarium with a clearly separated promentum (Fig. 4B).

In width, head = ring 4 = 5 < 6 < 7 < 3 < midbody rings (close to 8^th^ to 10^th^) < 2 < collum; body abruptly tapering towards telson on a few posteriormost rings (Fig. 3B). Postcollar constriction very evident (Fig. 3B).

Collum nearly smooth, carinotaxic formula 1–6+7a+pc+ma (Fig. 3A–C), with 7+7 longitudinal crests starting from anterior edge, but both median crests interrupted in about caudal 2/3–3/4, being replaced there by similar 1+1+1 crests.

Following metaterga similarly strongly crested (Fig. 3A, B, D–H), especially from ring 5 on, whence porosteles commence (Fig. 3A, B), smaller tubercles in their stead on legless rings in front of telson due to loss of ozopores (Fig. 3G, H). Porosteles large, conical, round, directed caudolaterad, wider than high. Midway metatergal crests on ring 5 distinctly divided into two at about 1/3 metatergal height, each half evident and well rounded, nearly undivided small tubercles in their stead in legless rings in front of telson (Fig. 3G, H). Carinotaxic formulae 3+I/i+3/3+I/i+3 on rings 2–4, as well as on the last one or two leg-bearing, and on legless rings (Fig. 3A, B, G, H); midbody rings showing mostly dorsal crests distinctly divided into two at about 1/3 metatergal height, each half rather evident and well rounded (carinotaxic formulae 3/3+I/i+4/3+I/i+3/3) and sharper, especially so lateral crests (Fig. 3D, E).

Tegument rather smooth, dull throughout (Fig. 3A, B, D, E, G, H). Fine longitudinal striations in front of stricture between pro- and metazonae, remaining surface of prozonae very delicately shagreened (Fig. 3D, E). Metatergal setae absent. Rings 2 and 3 each with long pleural flaps (Fig. 3A). Epiproct (Fig. 3G, I) simple, regularly rounded caudally, faintly convex medially. Paraprocts regularly convex, each with premarginal sulci medially and a row of sparse setae at medial margin (Fig. 3I). Hypoproct transversely bean-shaped, slightly concave caudally, with 1+1 strongly separated setae near caudal margin (Fig. 3I).

Ventral flaps behind gonopod aperture on ♂ ring 7 distinguishable as low swellings forming a bare transverse ridge.

Legs rather short, on midbody rings about half the length of ring height (Figs 3A, C, E–G, 4G). Claw at base with an evident accessory spine about 1/3–1/4 the length of main claw (Fig. 4G).

♂ legs 1 highly characteristic (Fig. 4C, D) in being very strongly reduced, represented only by a sternum devoid of any median or paramedian structures, but carrying 1+1 strongly separated prongs, both evidently curved posteriad and bearing several strong setae, and rudimentary, 1-segmented leg vestiges at base on caudal face (Fig. 4C, D).

♂ legs 2 nearly normal (Figs 4E), prefemur somewhat reduced only anteriorly; penes broad, oblong-subtrapeziform, each with 3–5 strong setae distolaterally (Fig. 4E).

♂ legs 3 modified in having coxa especially slender and elongate (Fig. 4F).

Anterior gonopods (Fig. 4H, J) with a typical shield-like coxosternum, the latter moderately microsetose in anterior and medial parts on caudal face (Fig. 4H), with a high, digitiform, apicomesal process (d). Telopodite rather small, movable, 1-segmented, lateral in position, with two or three strong apical setae and a field of microsetae at base (Fig. 4H), moderately higher than adjacent lateral corner of coxosternum.

Posterior gonopods (Fig. 4J, K) compact, broadly subquadrate, micropapillate medially on oral face; coxite medio-apically with a long, plumose, apical flagellum (f) with evident spikes paramedially (Fig. 4J, K); lamelliform lobe (l) high, subquadrate, membranous, wrinkled frontolaterally, with an apical field of coniform microsetae laterally (Fig. 4K); each mediolateral part of coxite with 10–12 strong and curved setae (Fig. 4K).

##### Remarks.

The *granulatus*-group currently encompasses 36 described species, including our new species (Golovatch et al. 2007a, 2011a; Likhitrakarn et al. 2017; Liu and Wynne 2019). The new species is the fifth in this group to be reported from Thailand.

Three populations have been collected inside caves, with the longest distance of about 25 air-km between the collecting localities, and all show similar morphological characters as described above. *Glyphiuluspromdami* sp. nov. fails to show any morphological adaptations to cave life and is considered here as a troglophilic species. It appears to have a rather narrow distribution, but has been found in a wide range of cave environments from the twilight (Tham Chetawan) to the dark and deep zones of the caves (all three caves). The temperature in the caves where the species was collected ranged between 24.2 and 29.8 °C, while the relative humidity was 70–94%. All populations were found to be quite large and associated with bat guano.

#### ﻿Key to *Glyphiulus* species presently known to occur in Thailand, based mainly on male characters

**Table:** 

1	First male legs either normal or reduced in size, but with a pair of paramedian coxal processes (Fig. 2C, D)	**2 (the *javanicus*-group)**
–	First male legs very strongly reduced, completely lacking any median structures (Fig. 4C, E)	**3 (the *granulatus*-group)**
2	Carinotaxic formula of collum, 6+6, all complete crests. Each anterior gonopod with a typical, digitiform, apicomesal process (d) (Fig. 4H, I). Each posterior gonopod with a short and bare flagellum	*** G. siamensis ***
–	Carinotaxic formula of collum, 1+2a+3c+4–5+6c+7a+pc+ma (Fig. 1A, B). Each anterior gonopod with a very long, slender, apicomesal process (d) (Fig. 2G, H). Each posterior gonopod with a very long, medial, densely plumose flagellum (Fig. 2I, J)	***G.longus* sp. nov.**
3	Body usually larger: length 23–40 mm. Carinotaxic formula of midbody rings, 2/2+I/i+4/3+I/i+2/2	*** G. sattaa ***
–	Body usually smaller: length 14–24 mm. Carinotaxic formula of midbody rings, 3/3+I/i+4/3+I/i+3/3	**4**
4	Carinotaxic formula of collum, 1–4+5+6c+7a+pc+ma. Ocelli unpigmented, mostly invisible, only sometimes traceable as light greyish, with 6–8 translucid ocelli. Legs long, about 1.1–1.2 the length of ring height	*** G. mongkon ***
–	Carinotaxic formula of collum different. Ocelli pigmented, with 8–16 blackish ocelli either side of head. Legs short, about 0.5–1.0 the length of ring height	**5**
5	Carinotaxic formula of collum, 1a+2–5+pc+ma. Telopodites of first male leg-pair two-segmented	*** G. duangdee ***
–	Carinotaxic formula of collum, 1–6+7a+pc+ma (Fig. 3A, B). Telopodites of first male leg-pair one-segmented (Fig. 4C, D)	**6**
6	14–16 ocelli in three or four irregular transverse rows. Each anterior gonopod with a very short apicomesal process (d), the latter as high as telopodites	*** G. maidtreejit ***
–	8–13 ocelli in three or four irregular transverse rows, always black. Each anterior gonopod with an elongated apicomesal process (d), the latter obviously higher than telopodites (Fig. 4H, I)	***G.promdami* sp. nov.**

## ﻿Discussion

The millipede family Cambalopsidae is known to be the most common, highly abundant and species-rich group dominating the cave diplopod faunas of Southeast Asia and China (Golovatch 2015). *Glyphiulus* is the largest genus and it presently comprises 68 described species. Most of the species (43, or > 63%) have been recorded from southern China, followed by Vietnam (nine species), northern Thailand (seven species), Laos (six species), Japan (the Ryukyu Islands) and Indonesia (north-central Java) (one species each) (Golovatch et al. 2007a, b; 2011a, b, 2012a; Likhitrakarn et al. 2017; Jiang et al. 2017, 2018, 2020; Liu and Wynne 2019). However, the diversity of this genus in Thailand is incompletely assessed, with only a small area having yet to be revealed as being confined to the northern, mountainous parts of the country (Fig. 5).

Only five *Glyphiulus* species have been found, and four described from caves, in Thailand: *G.duangdee* Golovatch, Geoffroy, Mauriès & VandenSpiegel, 2011b, *G.maidtreejit* Golovatch, Geoffroy, Mauriès & VandenSpiegel, 2011b, *G.mongkon* Golovatch, Geoffroy, Mauriès & VandenSpiegel, 2011b, and *G.sattaa* Golovatch, Geoffroy, Mauriès & VandenSpiegel, 2011b (Table 1). Now the diversity has been increased to seven, considering both new species documented in this study. On the other hand, since Thailand and the neighboring countries are known to be extremely rich in karst and caves, there can hardly be any doubt that additional species of *Glyphiulus* still await discovery. In contrast to the distribution of cave *Glyphiulus*, which is restricted to northern Thailand with all species being narrowly endemic, the similarly mostly cavernicolous genera *Plusioglyphiulus* and *Trachyjulus* are widespread throughout Thailand, while their species are likewise mostly narrowly endemic.

Most species of *Glyphiulus* in Thailand are known from caves or surrounding karst areas, except for *G.siamensis* Mauriès, 1983 which has been recorded from Kontathan (= Montha Than) waterfall area, Doi Suthep National Park, Chiang Mai Province (Mauriès 1983; Enghoff 2005). Similarly, *G.formosus* (Pocock, 1895) has been found and redescribed from under stones in a typical deciduous forest habitat in Guangdong Province, China (Jiang et al. 2020). This is evidence that many more cambalopsids can also be expected to be revealed outside caves, as they also live epigeically on the forest floor. Still, because locating millipedes inside caves or grottos on guano seems to be easier than searching for them in forest litter or tree stumps, the obvious bias to spotting “cavernicolous” Cambalopsidae is easy to comprehend. There is little doubt that, with further study of the millipede fauna of Thailand, both epigean and cavernicolous, many more novelties are to be expected.

## Supplementary Material

XML Treatment for Glyphiulus longus

XML Treatment for Glyphiulus promdami
